# Supplementary material for: Breast Milk Content of Vitamin A and E from Early- to Mid-Lactation Is Affected by Inadequate Dietary Intake in Brazilian Adult Women
Source: Nutrients. 2019 Aug 29;11(9):2025. doi: 10.3390/nu11092025 (PMC6770016; doi:10.3390/nu11092025)
Supplement: Supplementary file 1 [file nutrients-11-02025-s001.zip › Supplementary_Table_nutrients-555928.docx]

Breast milk content of vitamin A and E from early- to mid-lactation is affected by inadequate dietary intake in Brazilian adult women. M. R. Machado *et al*.

**Supplementary Table 1.** Calculated sample size necessary to disclose differences between early- and mid-lactating women in the distribution of vitamin A and E, and carotenoids in HDL and LDL+VLDL serum fractions.

| Vitamin or carotenoid | Calculated sample size (*n*)* | |
| --- | --- | --- |
|  | HDL | LDL + VLDL |
| Retinol | 18,505 | 38,205 |
| α-Tocopherol | 497 | 497 |
| γ-Tocopherol | 64 | 75 |
| Lutein + Zeaxanthin | 92 | 109 |
| β-Carotene | 1,162 | 2,493 |
| α-Carotene | infinity | infinity |
| Lycopene | infinity | infinity |

* for a pre-specified α of 0.05 (assuming *p* < 0.05 would be considered significant, in two-tailed comparisons) and a pre-specified β (power) of 0.80.
