# Supplementary figures and images for: Breast Milk Content of Vitamin A and E from Early- to Mid-Lactation Is Affected by Inadequate Dietary Intake in Brazilian Adult Women
Source: Nutrients. 2019 Aug 29;11(9):2025. doi: 10.3390/nu11092025 (PMC6770016; doi:10.3390/nu11092025)

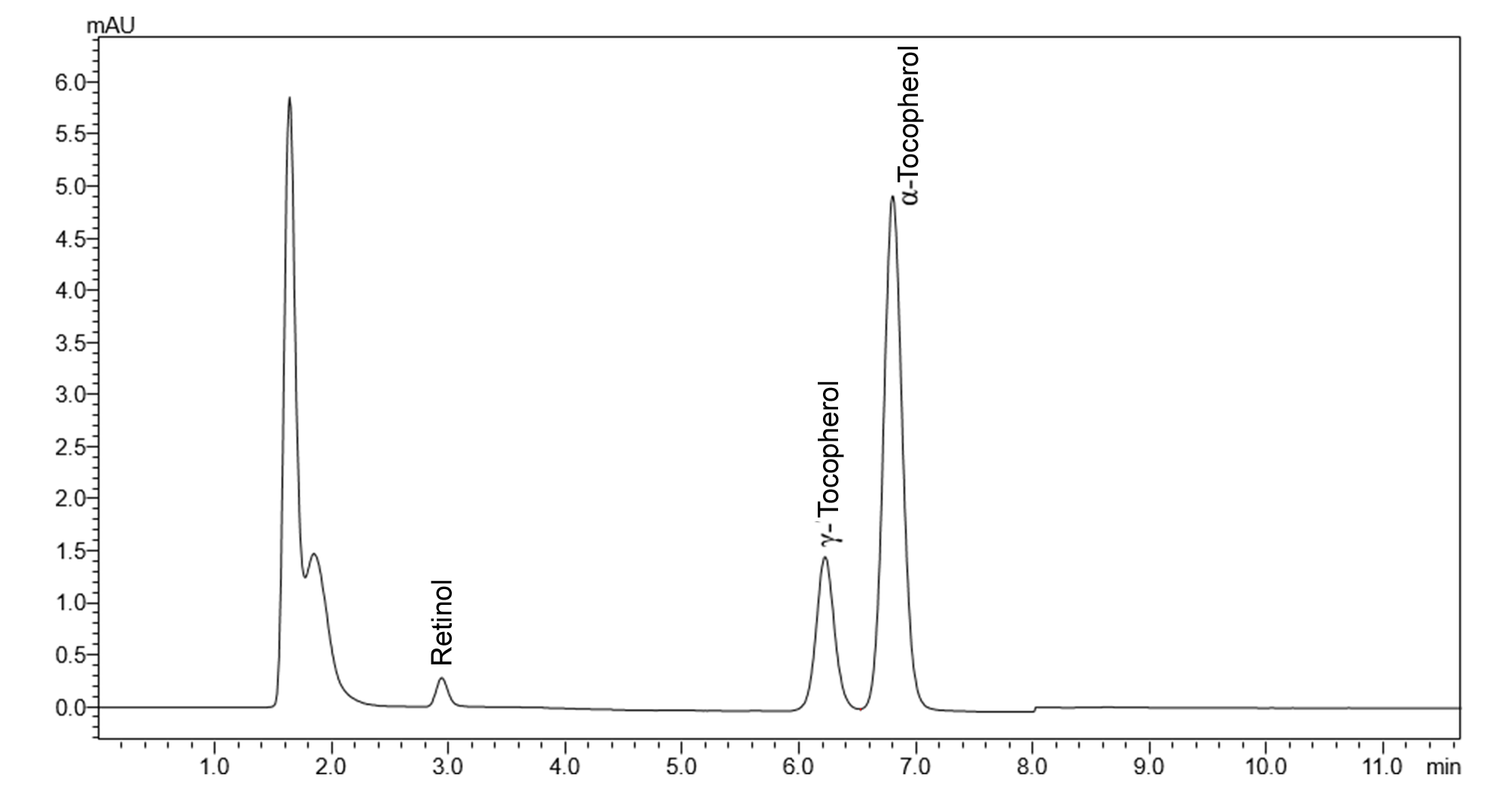

Supplement: Supplementary file 1 [file nutrients-11-02025-s001.zip › Figures/Supplementary_Fig1A.tif]

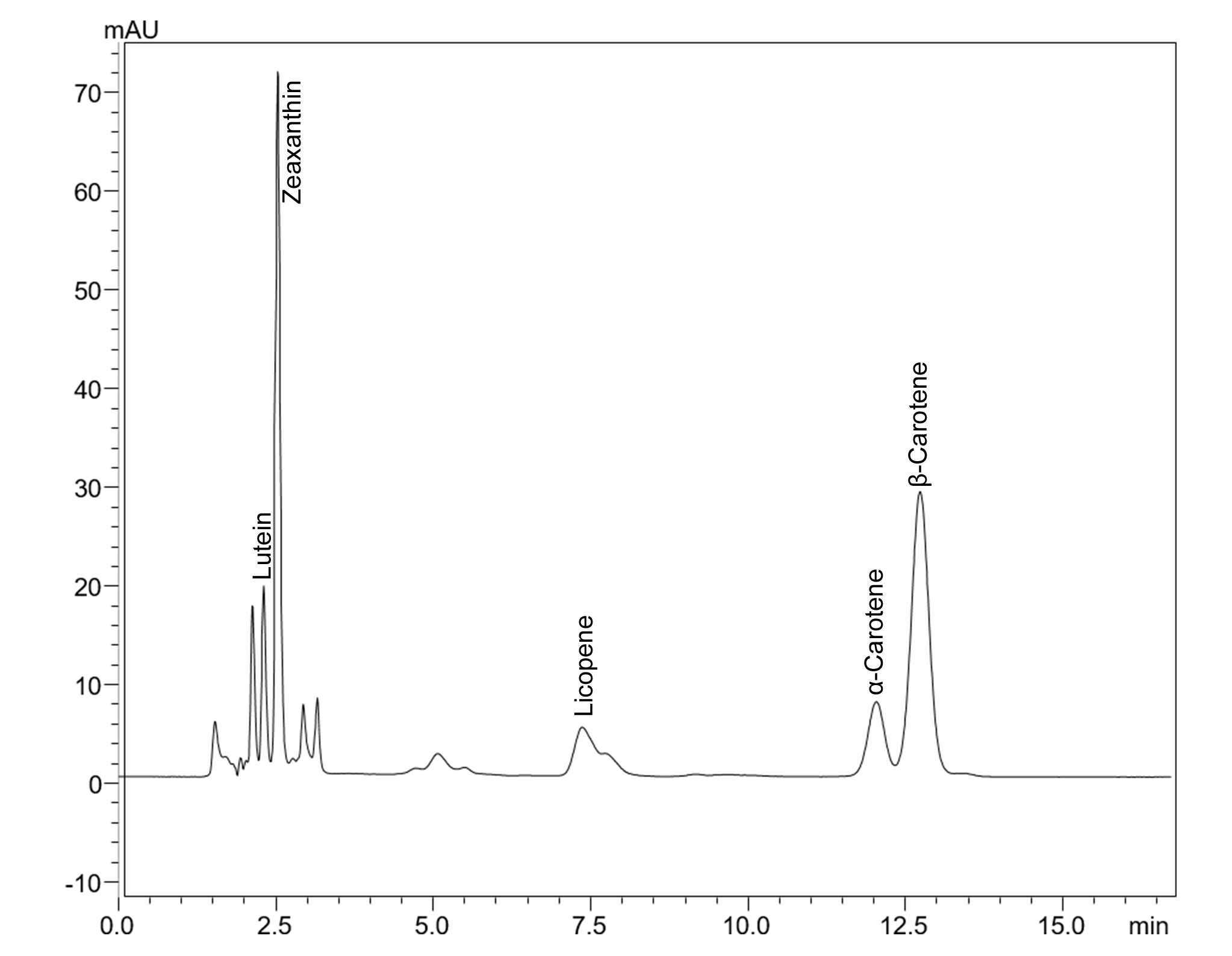

Supplement: Supplementary file 1 [file nutrients-11-02025-s001.zip › Figures/Supplementary_Fig1B.tif]
